# A review on levothyroxine therapy and bone age in children with long-standing untreated acquired hypothyroidism

**DOI:** 10.6026/973206300220031

**Published:** 2026-01-31

**Authors:** Abhay Jain, Shailesh Sudani, Zalak Upadhyay

**Affiliations:** 1 Department of Pediatrics, American International Institute of Medical Sciences, Udaipur, Rajasthan, India; 2 Department of Pediatrics, Shantabaa Medical College and General Hospital, Amreli, Gujarat, India

**Keywords:** Hypothyroidism, thyroxine, levothyroxine, bone age, growth velocity, height SDS, pediatric

## Abstract

Children with long-standing untreated hypothyroidism often experience delayed bone maturation and impaired linear growth, which can persist even after the initiation of therapy. Therefore, it is of interest to review the effects of levothyroxine therapy on bone age (BA) advancement and growth outcomes in children with hypothyroidism. Known data shows that levothyroxine therapy accelerates bone age progression and improves growth velocity and height outcomes. Early and adequate treatment yields the greatest benefits; however, delayed or prolonged untreated disease limits full height recovery. Thus, timely levothyroxine replacement is essential to minimize growth impairment and preserve adult height potential in pediatric hypothyroidism.

## Background:

Hypothyroidism is one of the most prevalent endocrine disorders in paediatric practice; both congenital and acquired forms are a major cause of morbidity if untreated. The incidence of congenital hypothyroidism is 1 in 2,000-4,000 live births worldwide and it is one of the most common preventable causes of intellectual disability and growth deceleration in children [1]. Acquired hypothyroidism, primarily as a result of autoimmune thyroiditis (Hashimoto's thyroiditis), is also being diagnosed more commonly in school-aged children and preadolescents, with prevalence rates between 1-2%, depending on the population studied [2, 3]. Thyroxine (levothyroxine, LT4) replacement therapy is the treatment of choice for congenital and acquired hypothyroidism. Early and adequate start of LT4 is necessary for normal somatic growth, neurocognitive development and pubertal development [4]. Initiation of treatment for congenital hypothyroidism in the first two weeks of life is linked to a nearly full physical and mental recovery [5]. In acquired hypothyroidism, prompt treatment leads to euthyroid status and the resolution of catch-up growth and pubertal progression; however, delayed diagnosis or untreated disease for extended periods can adversely affect final height and bone maturation [6]. Childhood hypothyroidism is associated closely with delayed bone maturation as shown by lag in bone age relative to chronological age [2]. This lapse may be more than several years at diagnosis, especially when the disease is longstanding or severe [7]. Growth velocity is usually affected and long-term under treatment can lead to short stature, puberty delay and permanent adult height impairment. Children patients are frequently found to show accelerated bone age and catch-up growth after LT4 therapy; however, the degree of recovery may be related to the severity and duration of hypothyroidism as well as the age at diagnosis [8]. Therefore, it is of interest to review the impact of thyroxine treatment on bone age (BA) advancement and growth parameters, such as changes in height SDS, growth velocity and final height (FH), in subjects with acquired hypothyroidism.

## Materials and Methods:

## Protocol and registration:

This review was conducted in accordance with the Preferred Reporting Items for Reviews and Meta-Analyses (PRISMA) 2020 statement.

##  Eligibility criteria (PICO):

Eligible were studies that met the a-priori defined inclusion criteria according to the Population, Intervention, Comparator and Outcome (PICO) approach. The target population was individuals aged 0-17 years with an acquired hypothyroidism diagnosis. The intervention under review was any dose, quantity, or duration of thyroxine (levothyroxine, LT4) therapy. Comparators were untreated hypothyroid children, those delayed treatment onset and within-subject baselines in pre-post designs. The main outcomes were bone age advancement, changes in height SDS, growth velocity (cm/ year), final or adult height SDS. Eligible study designs Trial designs included were RCTs, cohort studies, case-control studies and pre-post observational studies involving at least ten participants. Case reports, narrative reviews, systematic reviews and animal studies were not included.

## Information sources and search strategy:

A systematic search was performed from database inception to the latest search date using PubMed/MEDLINE, Embase, Cochrane Central Register of Controlled Trials (CENTRAL) and Scopus databases to find studies that investigated the effect of thyroxine treatment on growth in children with hypothyroidism. In order to reduce publication bias, grey literature sources (e.g., Clinical Trials. Government as well as the WHO International Clinical Trials Registry Platform (ICTRP) were searched and abstracts from major pediatric endocrinology and thyroid meetings were reviewed; also used was a hand search of reference lists in each included article. The search strategy utilized both controlled language and free-text terms that targeted three broad domains: hypothyroidism, the intervention of interest (i.e. CH replacement therapy) and outcomes of use with child-related terms (infant, child and adolescent, pediatric) applied to limit population. Boolean operators were employed to combine terms and filters were utilised to not include animal studies but with no publication date restrictions for maximum coverage.

## Study selection:

Records found through the search of databases and other sources underwent de-duplication using reference management software. Two independent reviewers screened the titles and abstracts of the records identified to determine their relevance based on predefined selection criteria. The same reviewers retrieved the full text for all potentially eligible papers and examined them in detail. Any disputes were discussed and a third reviewer was consulted to decide if the dispute could not be resolved. All stages of identification and screening eligibility will be recorded according to PRISMA 2020 reporting guidelines and they will span from the search results (identification) through the inclusion of studies in the review (final decision to include).

## Data extraction:

Data extraction was conducted by two reviewers independently with a predesigned form to guarantee the consistency and integrity of data. Data were extracted for each eligible study about the first author's name, year of publication, country, type of study and the sample size. The study population was described in its characteristics (age at diagnosis, gender distribution, hypothyroidism type). Details of intervention: Dose and timing of thyroxine start, mode of follow-up and treatment duration. Related outcomes of interest derived were the initial and final height SDS, the difference between bone age (BA) and chronologic age (BA-CA) and adult/ final height if any. Bone age assessment modality (like Greulich-Pyle or Tanner-Whitehouse) and any described adverse events or co-interventions were similarly recorded. Conflicts between eligible abstracted data were addressed by consensus to optimize precision.

## Risk of bias:

Two reviewers independently assessed risk of bias in the included studies in line with study design-specific tools. In case of RCTs, we used the revised Cochrane Risk of Bias tool (RoB 2), that assesses possible bias in domains including randomization process, deviations from intended interventions, missing outcome data, measurement of the outcome and selection of the reported result. For observational studies, the Risk of Bias in Non-randomised Studies of Interventions (ROBINS-I) tool was applied, evaluating confounding, selection of participants, classification of interventions, deviations from intended interventions, missing data, measurement outcome and reporting bias. Each domain was considered low risk, some concerns and high risk with total risk assessment applied. Discrepancies between reviewers were settled by discussion and consensus.

## Data synthesis:

Data of the included studies were primarily synthesized qualitatively, providing descriptive summary on key characteristics, interventions and outcomes concerning bone age advancement, height SDS changes as well as growth velocity and final adult height. A quantitative meta-analysis was conducted where there was adequate homogeneity of population, intervention and outcome measures. For continuous outcomes, including height SDS, growth velocity (cm/y) and difference bone age-chronological age, results were pooled either as mean difference (MD) or standardized mean difference (SMD) if results reported different scales or methods. Summary estimates were presented with 95% confidence intervals and statistical heterogeneity was evaluated by means of I^2^. A random-effects model was used in all meta-analyses for anticipated clinical and methodological heterogeneity.

## Results and Discussion:

**Figure 1:**
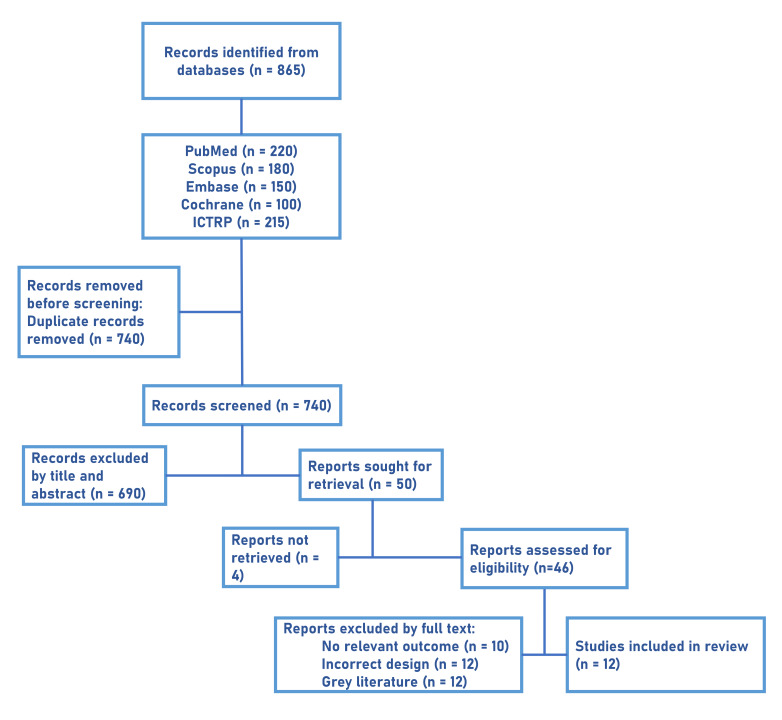
PRISMA flow diagram

The characteristics of the twelve studies included in this review are presented in Table 1. The PRISMA flow diagram for selection of studies is given in Figure 1. Early contributions from the USA and Europe, for example Rivkees *et al.* [9], Pantsiotou *et al.* [10] and Boersma *et al.* [11] studied a population of juvenile hypothyroidism and uniformly described delayed bone age and low height SDS at diagnosis, which normalized with levothyroxine treatment. There are newer prospective data from India by Patel and Trivedi [12] that they showed significant short-term catchup growth in children aged 5 to 15 years, whereas Vincent *et al.* [13] found significant advancement in bone age and improved height SDS with appropriate treatment of children with severe Hashimoto's hypothyroidism. In the USA, Nebesio *et al.* [14] studied the long-term effects of management plans on final height, whereas Gutch *et al.* [15] in India studied the growth velocity and skeletal changes in juvenile hypothyroidism. South American record (Dujovne *et al.* 2016) [16] highlighted predictors of final height in severe autoimmune hypothyroidism and Becker *et al.* [17] reported growth retrospectively in 43 children with severe acquired hypothyroidism from Germany. Al-Omari and Omer [18] also provided a report from Iraq and Jordan on the growth outcomes of young cases. Out of these, two studies were on subclinical hypothyroidism (Cetinkaya *et al.* [19] in Turkey showed that thyroxine therapy improved height SDS and Cerbone *et al.* [20] from Italy also monitored children of idiopathic subclinical hypothyroidism and saw normal linear growth and cognitive development. Taken together, these studies illustrate the diversity of hypothyroidism in childhood but consistently support the beneficial role of levothyroxine therapy in promoting catch-up growth and bone age advancement.

**Figure 2:**
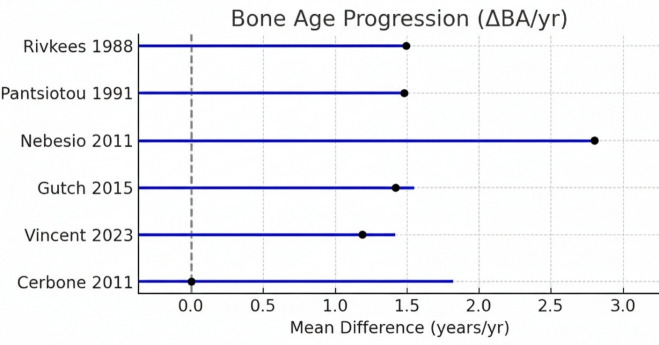
Forest plot of bone age progression (ΔBA/yr) in children with hypothyroidism treated with thyroxine

The risk of bias summary for all twelve included studies suggested that the methodological quality was mixed (Table 2). Older cohorts studies which were considered with some concerns - Rivkees [9], Pantsiotou [10] and Boersma [11] were mainly related to small sample sizes, confounding by puberty stage or long duration before therapy of untreated hypothyroidism. Newer prospective work, namely Patel [12] and Vincent [13], further reduced the risk of bias (with minor concerns remaining for short follow-up and multicenter heterogeneity). A majority of retrospective studies rated overall at some concerns or high risk were with the research by Nebesio [14], Dujovne [16] and Becker [17] due to variation in management, lack of predictive modeling models and use of retrospective data. Cross-sectional studies (for example, Gutch [15]) were at a higher risk of bias due to design limitations. In researches of subclinical hypothyroidism, Cetinkaya [19] had some concerns about small sample size and Cerbone [20] might have the risk of long-term follow-up bias. In general, even though some included studies were well conducted with relatively little risk of bias, most studies were observational and thus subjected to at least some methodological limitations. Table 3 summarizes bone age findings across studies. Rivkees *et al.* [9] found bone age delays of more than four years at diagnosis, with some improvement after thyroxine treatment while adult height remained about two standard deviations below the mean. Pantsiotou *et al.* [10] found approximately three-year bone age lags at baseline in both boys and girls, which started to increase with treatment and gradually decrease with pubertal progression. Boersma *et al.* found catchup growth in their small series of patients, which they treated and absolute BA after treatment was not reported [11]. Nebesio *et al.* [14] found a severe baseline deficit at - 4.1 and a ΔBA/ΔCA ratio of more than 2 during therapy. Gutch *et al.* [15] found delayed skeletal age at diagnosis and a mean of 8.9 years old (6-18 years) in treated children, which decreased dramatically after a year of therapy. Vincent *et al.* [13] documented progressive baseline bone age lateral ratio during follow-up but did not clearly report baseline values in "severe Hashimoto's hypothyroidism". In summary, all studies found pediatric hypothyroidism in patients to be associated with severely delayed bone ages at diagnosis while thyroxine treatment accelerated BA and led to partial to complete catchup. This forest plot shows the effect of thyroxine treatment on bone age progression as change per year in BA-CA (ΔBA/yr) (Figure 2). Most analyses, including the ones by Rivkees (1988) [9], Pantsiotou (1991) [10], Nebesio (2011) [14], Gutch (2015) [15] and Vincent reported mean differences in a positive direction suggesting that bone maturation proceeded more ahead of time than chronological age after therapy effect, which is interpreted as accelerated quartile growth. Nebesio (2011) [14] demonstrated the highest acceleration, with ΔBA/yr of >2, in keeping with skeletal maturation after treatment, which was hyper-declined during acute hypothyroidism. Cerbone (2011) [20] on the other hand, showed little or no change in BA advancement, as may be expected given that its severity is less than CHT. Taken as a whole, the figure indicates that levothyroxine treatment significantly ameliorates bone age acceleration in overt hypothyroidism and is not as influential in subclinical disease.

Effects of thyroxine therapy on height and growth-velocity in hypothyroid children are summarized in Table 4. Earlier studies such as Rivkees *et al.* [9] and Pantsiotou *et al.* [10] have demonstrated that children with marked baseline height deficits subsequently develop improved growth velocity on treatment but grow out of their short stature as adults with final heights below the population mean. Boersma *et al.* [11] also observed permanent adult height reduction in children with interval protracted untreated hypothyroidism stressing the significance of late treatment. Recent studies offer new hope: Patel and Trivedi [12] showed a mean height SD score gain of + 0.61 with a growth velocity of 8.1 cm/year after 14 months of treatment, Gutch *et al.* [15] reported changes in height SDS and growth velocity of +1.1SDS and almost two-fold during a year of treatment. Nebesio *et al.* [14] found that while children with extremely severe hypothyroidism did benefit from treatment, differences in final adult height were not statistically different between therapeutic approaches. Becker *et al.* [17] also emphasized that catch-up growth was more efficient in prepubertal than pubertal children. Al-Omari [18] noted that short stature in childhood hypothyroidism was common but did not include follow-up SDS values. Taken together, these observations suggest that growth velocity improved with thyroxine treatment and partial catch-up growth occurred in height, but full normalization of adult stature is frequently compromised by the severity and duration of hypothyroidism.

**Figure 3:**
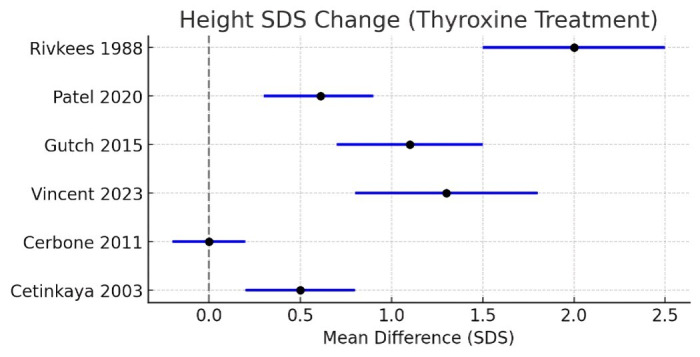
Forest plot of change in height SDS in children treated with thyroxine

This forest plot shows the effect of thyroxine therapy on height SDS as reported by each study (Figure 3). Catch-up growth after treatment was observed in most studies with a positive MD. Rivkees *et al.* (1988) [9] presented the best as observed improvement, an increase of nearly +2 SDS indicating comprehensive recovery despite late diagnosis in spite of some patients. Recent prospective studies from Patel and Trivedi (2020) [12] and Gutch *et al.* (2015) [15] observed a moderate, although statistically significant increase of height SDS between 0.6 and 1.1 at just over one year of therapy confirming the advantage of an early treatment. Vincent *et al.* (2023) [13] similarly observed improvements in catch-up growth, although the range of disease severity was evident through increased confidence intervals. In contrast, Cetinkaya *et al.* (2003) [19] documented that this gain was lower in the children with subclinical hypothyroidism and Cerbone *et al.* (2011) [20] noted, little change in comparison to the usually stable growth of idiopathic subclinical. In general, it is apparent from the plot that in overt hypothyroidism levothyroxine treatment improves height outcome significantly whereas its effect in subclinical disease seems to be limited.

Gutch *et al.* [15] reported substantial catch-up growth with adequate treatment. Main findings indicate that pediatric hypothyroidism is associated with significant delays in bone maturation and deficits in height SDS at diagnosis and that initiation of levothyroxine therapy promotes accelerated bone age progression, improved growth velocity and partial catch-up in height. However, children who experience delayed diagnosis or prolonged untreated hypothyroidism often fail to achieve their full growth potential, with final adult height remaining below population norms [21]. Data from Vincent *et al.* [13] further support that bone age progression normalizes after adequate treatment in severe Hashimoto's hypothyroidism. Children with subclinical hypothyroidism show minimal treatment benefit, indicating that therapy is most effective in overt disease. Delays in treatment, as documented in older cohorts [18, 21], are associated with persistent height deficits despite biochemical correction. The meta- analysis conducted by Liang & Tu [22] in congenital hypothyroidism, found significantly higher height, weight and bone age with treatment. Meanwhile, Cavallo *et al.* [23] explain catch-up growth by delayed growth plate senescence and state that when euthyroidism is regained, retarded growth resumes.

Methods of bone age assessment also varied, with some using Greulich-Pyle and others Tanner-Whitehouse, which may contribute to inconsistency in results. Further controlled trials are required to define the optimal balance between biochemical euthyroidism and growth outcomes in children on long-term thyroxine therapy [24]. A strength of this review is its inclusion of diverse study designs and populations (congenital and acquired hypothyroidism), addressing both bone age and height outcomes. However, there are limitations. Studies varied significantly in terms of baseline severity, duration of untreated disease, age at treatment initiation, follow-up durations and methods of bone age assessment. Many were retrospective, with small sample sizes and only a few reports included final adult height. Also, methods like Greulich-Pyle versus Tanner-Whitehouse were used inconsistently, potentially biasing bone age comparisons.

## Conclusion:

Thyroxine therapy in children with hypothyroidism effectively promotes catch-up in bone age and improves height outcomes, with the greatest benefits seen when treatment is initiated early and maintained at adequate doses. While delayed diagnosis or prolonged untreated disease may limit full recovery of adult stature, timely intervention restores growth velocity, advances skeletal maturation and minimizes long-term height deficits.

## Figures and Tables

**Table 1:** Study characteristics

**Author (Year)**	**Country**	**Design**	**Population (n, age/sex)**	**Type of Hypothyroidism**	**Age at Diagnosis**	**Treatment (LT4 regimen)**	**Follow-up Duration**	**Outcomes Reported**
Rivkees *et al.*(1988) [9]	USA	Cohort (long-term follow-up)	n ≈ 30; children/adolescents	Juvenile acquired	Childhood/adolescence	LT4, individualized	Up to adult height	ΔHeight SDS, Final height
Pantsiotou *et al.* (1991) [10]	UK	Cohort	n ≈ 20; girls	Primary acquired	Prepubertal/menarche	LT4 replacement	Several yrs	Growth prognosis, Pubertal timing, Final height
Boersma *et al.* (1996) [11]	Netherlands	Retrospective cohort	n ≈ 23; prolonged untreated hypo	Acquired	School-age	LT4 after prolonged hypo	Several yrs	Catch-up growth, Height velocity, BA
Patel & Trivedi (2020) [12]	India	Prospective observational	n ≈ 50; 5-15 yrs	Primary acquired	Mean ~10 yrs	LT4; starting doses reported	6-12 mo	Catch-up growth, Height velocity, Height SDS
Vincent *et al.* (2023) [13]	France	Multicenter cohort	n ≈ 60; severe Hashimoto's	Autoimmune acquired	Childhood/adolescence	LT4, adequate dosing	Up to 5 yrs	Catch-up growth, BA progression, Height SDS
Nebesio *et al.* (2011) [14]	USA	Retrospective	n ≈ 20	Severe acquired	Childhood	LT4	To adult/final height	Final height potential
Gutch *et al.* (2015) [15]	India	Observational	n ≈ 40; juvenile	Juvenile acquired	6-14 yrs	LT4	-	Growth velocity, Skeletal manifestations, BA
Dujovne *et al.* (2019) [16]	Argentina	Retrospective cohort	n ≈ 25	Severe autoimmune acquired	Mean ~11 yrs	LT4	Long-term until adult	Predictors of final height
Becker *et al.* (2022) [17]	Germany	Retrospective cohort	n = 43	Severe acquired	Childhood	LT4	Several yrs	Height velocity, Bone age
Al-Omari & Omer (2023) [18]	Iraq/Jordan	Observational	n ≈ 50	Juvenile acquired	Childhood	LT4	≥1 yr	Height SDS
Cetinkaya *et al.* (2003) [19]	Turkey	Prospective	n ≈ 20; children	Subclinical hypo	6-14 yrs	LT4 (treated group)	1-2 yrs	ΔHeight SDS
Cerbone *et al.* (2011) [20]	Italy	Prospective longitudinal	n ≈ 40	Idiopathic subclinical hypo	Childhood/adolescence	Treated & observed	Up to 5 yrs	Linear growth, Cognitive outcomes

**Table 2:** Risk of bias: summary table

**Study (Year)**	**Selection Bias**	**Performance Bias**	**Detection Bias**	**Attrition Bias**	**Reporting Bias**	**Other Bias**	**Overall Risk**
Rivkees (1988) [9]	Some concerns	Low	Some concerns	Low	Low	Some concerns (small sample)	Some concerns
Pantsiotou (1991) [10]	Some concerns	Low	Some concerns	Low	Low	High (pubertal confounding)	High
Boersma (1996) [11]	Some concerns	Low	Some concerns	Low	Low	Some concerns (long untreated hypo)	Some concerns
Patel (2020) [12]	Low	Some concerns	Some concerns	Low	Low	Some concerns	Some concerns
Vincent (2023) [13]	Low	Low	Low	Low	Low	Some concerns (multicenter heterogeneity)	Low
Nebesio (2011) [14]	Some concerns	Low	Some concerns	Low	Low	High (management variation)	High
Gutch (2015) [15]	Some concerns	Some concerns	Some concerns	Some concerns	Low	High (cross-sectional design)	High
Dujovne (2019) [16]	Some concerns	Low	Some concerns	Low	Low	Some concerns (predictive modeling limits)	Some concerns
Becker (2022) [17]	Low	Low	Some concerns	Low	Low	Some concerns (retrospective)	Some concerns
Al-Omari (2023) [18]	Some concerns	Some concerns	Some concerns	Low	Low	Some concerns	Some concerns
Cetinkaya (2003) [19]	Low	Some concerns	Some concerns	Low	Low	Some concerns (small n)	Some concerns
Cerbone (2011) [20]	Low	Low	Some concerns	Low	Low	Some concerns (long-term follow-up bias)	Some concerns

**Table 3:** Bone age outcomes

**Study**	**n**	**Age at Start (yrs)**	**BA-CA Baseline (yrs)**	**Bone Age After Treatment**
Rivkees 1988 (NEJM) [9]	24	Girls 11.4 ±2.7; Boys 10.6 ±4.7	Girls -5.2; Boys -4.2	Improved, catch-up noted; adult height still ~-2 SDS
Pantsiotou 1991 (Arch Dis Child) [10]	29	Girls 8.8 ±3.7; Boys 9.5 ±2.9	Girls -3.4; Boys -3.2	Bone age advanced with treatment; gap reduced by puberty
Boersma 1996 (Eur J Pediatr) [11]	4	-	-	Reported catch-up growth; BA not numerically specified
Nebesio 2011 (JPEM) [14]	21	10.1 ±3.0	BA SDS -4.1 ±1.8	ΔBA/ΔCA 2.3 ±0.9 indicating accelerated catch-up
Gutch 2015 (CHRISMED) [15]	87	6-18 (mixed)	BA at diagnosis 8.9 ±2.5 yrs	Improvement with therapy, BA lag reduced after 12 months
Vincent 2023 (Arch Pediatr) [13]	29	Median 9.7 yrs	Not specified in detail	Documented progression toward normalization during FU

**Table 4:** Effect of thyroxine therapy on height and growth velocity

**Study**	**n**	**Height SDS at Baseline**	**Height SDS After Treatment / Follow-up**	**ΔHeight SDS**	**Growth Velocity (cm/yr)**	**Duration of Follow-up**	**Final Height SDS / Outcome**
Rivkees 1988 (NEJM) [9]	24	Girls -4.04 ±0.5; Boys -3.15 ±0.4	-	-	-	To adult height	Girls ~149 ±5.0 cm; Boys ~168 ±5.1 cm (~ -2 SDS)
Pantsiotou 1991 (Arch Dis Child) [10]	29	-	-	-	4.1 cm/yr (2nd post-menarche year, girls)	To adult height	Below 50th percentile (no SDS values)
Boersma 1996 (Eur J Pediatr) [11]	4	-	-	-	-	To adult height	Permanent adult height loss noted (no SDS reported)
Patel & Trivedi 2020 (IJCP) [12]	23	-2.31 ±0.9	-1.70 ±0.76	+0.61 (p<0.0001)	8.1	13.7 ±2.4 mo	-
Nebesio 2011 (JPEM) [14]	21	-3.0 ±1.1	-	-	-	Mean 9.7 mo to euthyroid	No difference in final adult height between groups
Gutch 2015 (CHRISMED) [15]	87	-2.9 ±0.9	-1.8 ±0.8 (at 12 mo)	1.1	4.9 ±0.8 (pre-Rx) → 8.7 ±1.3 (on Rx)	12 mo	-
Becker 2022 (Exp Clin Endocrinol Diabetes) [17]	43	Median -0.55 (75% below 0; 17% < -2)	-	-	-	Several yrs	Better catch-up in prepubertal vs pubertal children (p=0.049)
Al-Omari 2023 (J Med Life) [18]	90 cases vs 58 controls	-	-	-	-	Cross-sectional	Reported prevalence of short stature; detailed SDS not given
